# Phagocytosis of Apoptotic Cells Is Specifically Upregulated in ApoE4 Expressing Microglia *in vitro*

**DOI:** 10.3389/fncel.2019.00181

**Published:** 2019-05-03

**Authors:** Christiane Muth, Alexander Hartmann, Diego Sepulveda-Falla, Markus Glatzel, Susanne Krasemann

**Affiliations:** Institute of Neuropathology, University Medical Center Hamburg-Eppendorf, Hamburg, Germany

**Keywords:** apolipoprotein E, microglia, cytoskeleton, cell motility, phagocytosis, cell signaling, Alzheimer disease

## Abstract

Alzheimer’s disease (AD) is characterized by intracellular tau aggregates and extracellular deposition of amyloid-β (Aβ). The major genetic risk factor to develop AD is the Apolipoprotein E isoform 4 (ApoE4). ApoE4 may directly affect Aβ pathology, yet the exact role of ApoE4 in the progression of AD remains unclear. Although astrocytes are the main source of ApoE in brain tissue, other cell types might contribute to ApoE isotype-dependent effects. While ApoE expression does not play a relevant role in homeostatic microglia, we and others could recently show that ApoE expression is significant upregulated in disease-associated microglia including AD-mouse models and human AD. ApoE has been supposed to have an anti-inflammatory effect, with ApoE4 being less effective than ApoE3. However, ApoE-isotype specific effects on microglia function in disease have not been thoroughly investigated to date. In contrast to this, the role of ApoE2, the third most common major ApoE isoform, in neurodegeneration has not been characterized in detail, but it has been shown to delay the onset of disease in familial AD. To elucidate the differential roles of the three-major human ApoE isoforms on microglia function we each expressed the human ApoE isoforms in murine N9 microglia cells. We could show that ApoE4 specifically influences actin cytoskeleton rearrangement and morphology. In migration assays, ApoE4 significantly promotes cell motility. To quantify phagocytosis by microglia we established an uptake assay based on imaging flow cytometry. Although expression of ApoE4 led to significantly reduced uptake of Aβ in contrast to the other isoforms, we could show that ApoE4 specifically increased phagocytosis of apoptotic neuronal cells. Our findings show that ApoE4 intrinsically affects microglia physiology by upregulating motility and phagocytic behavior *in vitro* and may therefore specifically contribute to microglia dysregulation in AD.

## Introduction

Alzheimer’s disease (AD) is a neurodegenerative disease with an increasing risk in the elderly which is characterized by the intracellular deposition of tau aggregates and extracellular by Aβ plaque accumulation (Reiman et al., 2009; Jucker and Walker, 2011). While only a small percentage of all AD cases are familial with mutations in the amyloid precursor protein or Aβ processing proteins, most cases of AD are sporadic. However, beside of age, carrying the allele of the human ApoE4 is the major genetic risk factor to develop late onset or sporadic AD (Corder et al., 1993). Homozygous carriers of ApoE4 have a 12-fold increased risk for developing AD and a decrease in the age of onset (Corder et al., 1993; Roses, 1996; Yu et al., 2014). In humans, three major isoforms (ApoE2, ApoE3, ApoE4) exist, that differ in only one or two single amino acids. ApoE3 is homozygous in more than 60% of the population and therefore the most common isoform. In contrast to ApoE4 which increases the risk to develop AD, carriers of the ApoE2 isoform have a delay in the age of onset in familial AD (Velez et al., 2016). In the brain, ApoE is mainly produced by astrocytes. However, also other cell types might contribute to ApoE expression to much lesser degree (Pitas et al., 1987; Uchihara et al., 1995; Bu, 2009). How ApoE4 increases the risk to promote AD is actually not completely clear. ApoE has been shown to co-localize with Aβ and accelerates Aβ aggregation. ApoE limits accumulation of Aβ in an isoform dependent manner (Schmechel et al., 1993; Bales et al., 1997), suggesting it may have an effect on its clearance and prompting research on isoform-dependent inflammatory mechanisms. Much of the work that deals with the potential role of ApoE4 in AD examines its relationship to Aβ. It has already been observed a positive correlation between ApoE4 allele dose and Aβ plaques density in post mortem brain tissue of individuals with AD (Rebeck et al., 1993). Furthermore, it is widely believed that ApoE4 affects the production, clearance and aggregation of Aβ (Liu et al., 2013; Huynh et al., 2017). ApoE4 may also play an important role in tau pathogenesis, since a recent study in a murine model of tauopathy co-expressing human ApoE4 led to a significant increase in neurodegeneration compared to that seen with other ApoE isoforms (Shi et al., 2017). Although both, tau and Aβ, are central components of AD pathology, immune cells, especially microglia have been increasingly implicated to play important roles in disease progression in neurodegenerative diseases (Heneka et al., 2015).

Microglia are the innate immune cells of the brain. While ApoE is expressed at very low levels by adult microglia in the healthy brain (Butovsky et al., 2014), we and others could show that ApoE is significantly upregulated in dysregulated microglia in the diseased brain (Chiu et al., 2013; Krasemann et al., 2017). Resting microglia constantly monitor the environment with their processes to screen the brain parenchyma for pathogens or cell debris (Nimmerjahn et al., 2005). Upon different stimuli such as invading pathogens, cellular debris and protein aggregates, they quickly change from a resting to an activated status (Kreutzberg, 1996). Cell morphology could be changed to an amoeboid form, which improves their motility (Leu and Maa, 2003; Dibaj et al., 2010; Kettenmann et al., 2011). These features are important for migration, pathogen recognition and the modulation of immune response, including the release of cytokines and phagocytosis of various debris (Ransohoff and Perry, 2009). While microglia display a unique homeostatic molecular and functional signature in the healthy brain (Gautier et al., 2012; Butovsky et al., 2014), this is lost during activation or in disease progression (Butovsky et al., 2015; Holtman et al., 2015). Recent studies identified a common disease-associated microglial signature which is associated with an increase of mainly pro-inflammatory genes, while homeostatic genes are downregulated (Chiu et al., 2013; Hickman et al., 2013; Orre et al., 2014; Keren-Shaul et al., 2017; Krasemann et al., 2017). Interestingly, ApoE is one of the top upregulated genes in disease associated microglia. Therefore, ApoE might be a critical factor in the dysregulation of microglia cells by inducing a specific transcriptional program in neurodegenerative diseases (Krasemann et al., 2017). Interestingly, the ApoE2 haplotype seems to be associated with a reduced age-related expression signature in human microglia in aging, pointing to a protective effect specifically in microglia (Olah et al., 2018). However, it is currently not known, if human ApoE4 specifically contributes to intrinsic dysregulation of microglia in disease.

To determine the potential differential effects of the three major human ApoE isoforms on microglia phenotypes and functions, we generated the three different human ApoE expressing cell lines based on the microglia cell line N9. We could show that specifically ApoE4 increases direct and indirect migration. Moreover, we demonstrated that the isoform ApoE4 influences the microglial actin cytoskeleton. Since Lin et al. (2018) showed that microglia derived from induced pluripotent stem cells expressing ApoE4 showed decreased phagocytosis of Aβ than those expressing ApoE3, we also investigated this function in our model: we could confirm the decreased Aβ phagocytosis caused by ApoE4 expression in N9 cells. Interestingly, ApoE2 did not increase phagocytosis of Aβ. To assess the ability of ApoE isoforms to influence microglial phagocytosis of apoptotic cells, we quantified these phagocytic microglia using imaging flow cytometry. In contrast to the findings regarding phagocytosis of Aβ, here, ApoE4 expressing microglia displayed significantly increased phagocytosis toward apoptotic neuronal cells. Our results indicate that ApoE4 expression might intrinsically predispose microglial toward a dysregulated phenotype. ApoE4 might therefore also facilitate the switch from healthy to disease-associated microglia in AD.

## Results

### ApoE4-Expressing N9 Cells Display a Dysregulated Phenotype

**FIGURE 1:**
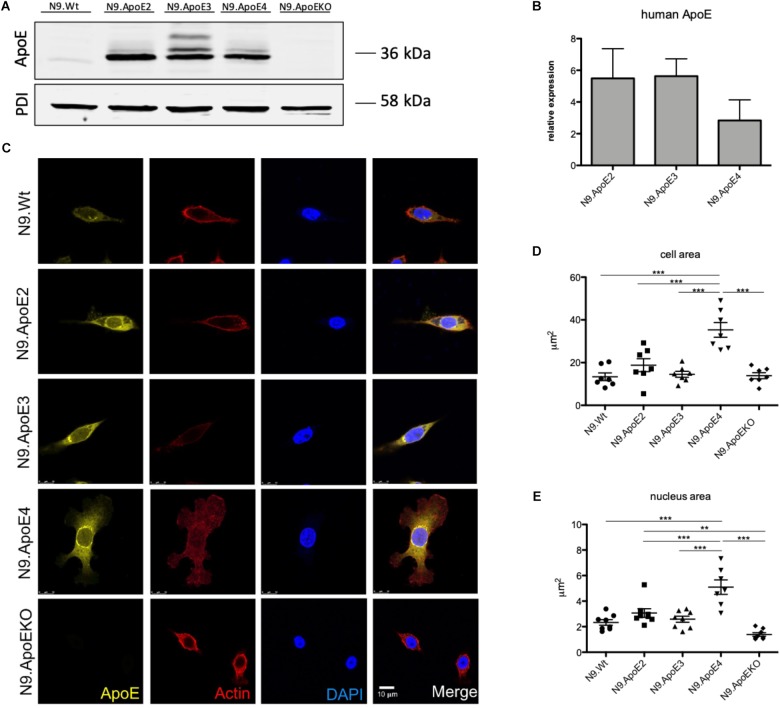
Microglia morphology is changed upon ApoE4 expression. **(A)** Western Blot analysis of the expression of the three human ApoE isoforms in the microglial cell line N9. PDI displays protein loading. N9.ApoEKO shows no expression of ApoE (*n* = 3). **(B)** qPCR analysis of human *ApoE* RNA-expression normalized against murine *GapDH* levels. Expression of human *ApoE* is only abundant in cells with human *ApoE* transduction and levels are comparable (*n* = 3). **(C)** Representative pictures of immunofluorescent imaging show changes in morphology of N9.ApoE4 and N9.ApoEKO. N9.ApoE4 demonstrates a significant increase in cell area **(D)**, and nucleus size **(E)**, whereby N9.ApoEKO displays a decrease in both. Additionally, many of N9.ApoE4 cells are much larger in comparison to the cells of the other cell lines (*n* = 7). Statistical significance was set up at ^∗∗^*p* < 0.01 and ^∗∗∗^*p* < 0.001.

To investigate the differential role of human ApoE isoforms on microglial physiology we each expressed one of the three major human ApoE isoforms in murine N9 microglia cells (designated as N9.ApoE2, N9.ApoE3, N9.ApoE4). Expression levels of murine *ApoE* is generally low in N9 cells and remained unchanged after transfection of the human isoforms when assessed by qPCR and western blot analysis (Figure 1A,B and Supplementary Figure 1A). RNA levels of the different human *ApoE* isoforms were comparable, although N9.ApoE4 showed a slightly reduced expression level in western blot analysis (not significant), which might be due to the fact that ApoE4 could be degraded faster than the other isoforms (Tamboli et al., 2014). Additionally, as a control, we generated a full ApoE knockout cell line (N9.ApoEKO) applying the CRISPR/Cas9-system (Figure 1A and Supplementary Figure 1A) which show no *ApoE* expression anymore. These cell lines were taken for further investigation.

To determine, if the major genetic risk factor to develop AD, ApoE4, leads to a more dysregulated/pro-inflammatory phenotype after inflammatory stimulation in N9 microglia cells, we perform gene expression analysis using NanoString technology. For this, we treated N9.ApoE3 and N9.ApoE4 cells with lipopolysaccharide (LPS), a potent toll-like receptor 4 (TLR-4) agonist. This treatment had been shown to induce isoform specific effects on cytokine production (TNFα, IL-6, IL12p40) in primary microglia derived from newborn humanized ApoE3 versus ApoE4 knock–in mice (Vitek et al., 2009). After 24 h of LPS treatment we analyzed changes in gene expression by directly measuring RNA counts using the NanoString mouse neuroinflammation panel. Expression of pro-inflammatory cytokines including *TNFα* was significantly increased after LPS treatment in N9.ApoE3 cells (Supplementary Figures 2A,B and Supplementary Table 1). However, ApoE3 versus 4 isoform specific changes after LPS treatment were confined only to a subset of the detected genes (Supplementary Figures 2C,D and Supplementary Tables 2, 3). These did not include *TNFα* or *IL-6*, but several other genes involved in pro-inflammatory signaling and cell stress. Moreover, expression of the disease marker *Spp1* was affected by ApoE-isoform and more upregulated in treated N9.ApoE4 cells (Supplementary Figure 2D). Of note, a transcription factor from the Mef-family, *Mef2c*, that we identified as key regulators affected by the ApoE pathway in dysregulated microglia *in vivo*, was also significantly affected by ApoE4 (Supplementary Figures 2C,D; Krasemann et al., 2017).

### ApoE4 Specifically Modifies Cell Morphology and Actin Cytoskeleton

The cell morphology is important for the regulation of many physiologically processes. Recently, Lin et al. (2018) showed altered morphologies of ApoE3 versus ApoE4 expressing iPSC-derived microglia-like cells. Therefore, we wanted to investigate if ApoE isoforms differentially influence the actin cytoskeleton of our microglia cell lines. Moreover, we wanted to assess, if ApoE2 specifically could counteract potential effects. We first examined cell morphology using confocal microscopy and observed a shift in cell shape in N9.ApoE4 and N9.ApoEKO cells compared to all others. The majority of N9.ApoE4 cells showed a significant increase in cell and nucleus area and displayed more discoidal shaped cells when compared to N9 wildtype (N9.Wt), N9.ApoE2 and N9.ApoE3 (Figure 1C–E). Of note, ApoE2 expression did not change the morphology of N9 cells. In contrast, only in N9.ApoE4 cells we observed distinct actin stress fibers together with an increase in expression of *Actin*, which we confirmed via immune fluorescence microscopy and qPCR (Supplementary Figures 1B,C). Actin stress fibers, cytoskeletal structures composed of contractile actin and myosin bundles, are another crucial aspect in regulating cell motility (Tojkander et al., 2012). If the structure of actin stress fibers is disturbed this might lead to an altered migration behavior of microglia cells (Pantaloni et al., 2001;Wehrle-Haller and Imhof, 2003).

**FIGURE 2:**
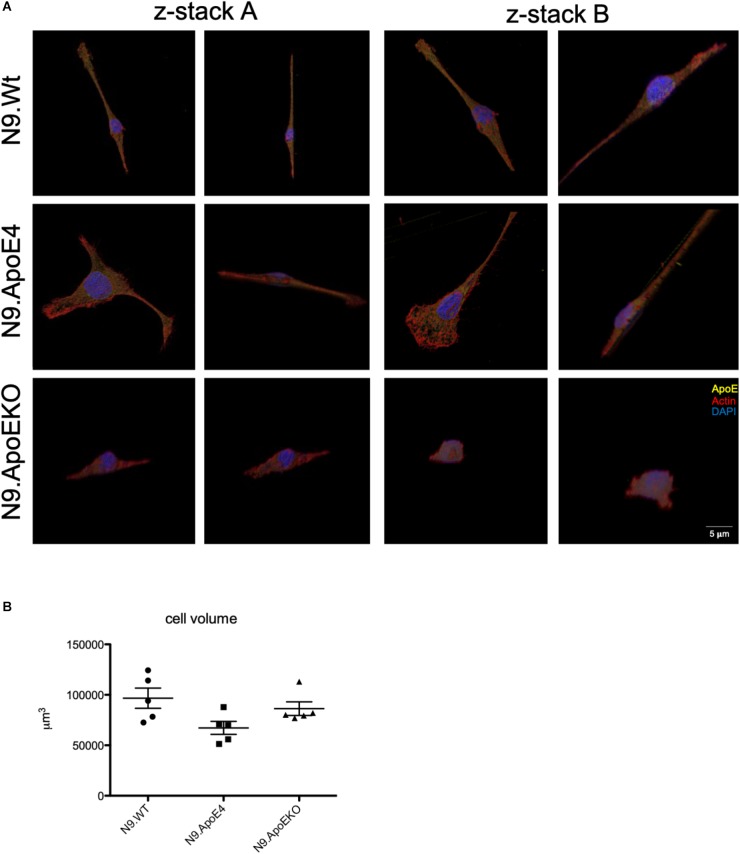
3D-reconstruction of z-stack images display discoidal morphology of N9.ApoE4 cells. **(A)** Two representative z-stack images from N9.Wt, N9.ApoE4, and N9.ApoEKO were reconstructed with IMARIS ×64 software. N9.ApoE4 show a more discoidal cell form in comparison to N9.Wt and N9.ApoEKO. In addition, N9.ApoE4 have distinct actin stress fibers (*n* = 5). **(B)** Quantification of cell volume demonstrate no significant differences in N9.ApoE4 and N9.ApoEKO in comparison to N9.Wt (*n* = 5).

As we only observed morphological differences in N9.ApoE4 and N9.ApoEKO cells, these were examined more closely. Applying 3D reconstruction we found that N9.ApoEKO displayed a reduction in cell volume in comparison to N9.Wt (Figure 2A). In contrast, N9.ApoE4 expressing cells showed increased cell area (Figure 1D) but no change in cell volume in comparison to N9.Wt (Figure 2B). Interestingly, cells were more flattened and discoidal in comparison to N9.ApoEKO, which showed a more round shape.

**FIGURE 3:**
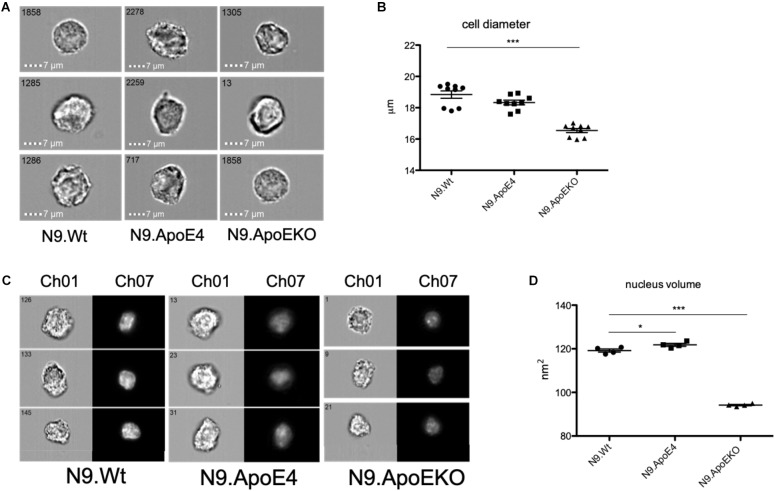
ApoE4 leads to an increase in nucleus volume and cell diameter. **(A)** Cell diameter of 5000 cells of each ApoE-isoform was measured at a magnification of 40×. N9.ApoE4 demonstrate an increase whereas N9.ApoEKO show a decrease in cell diameter in comparison to the other cell lines. **(B)** Quantification of cell diameter of five independent experiments (*n* = 5) was done with AMARIS software. **(C)** Representative images of the pictures taken with imaging flow cytometry. 5000 cells were measured at a magnification of 40×. Ch01: bright-field; Ch07: DAPI. **(D)** Quantification of nucleus volume with AMARIS software showed an increase in N9.ApoE4, whereby N9.ApoEKO display a decrease in comparison to N9.ApoE3 and N9.ApoE4 (*n* = 6). Statistical significance was set up at ^∗^*p* < 0.05 and ^∗∗∗^*p* < 0.001.

To independently quantify total cell diameter of the different human ApoE isoform expressing cells by an automated method and higher throughput, we performed imaging flow cytometry. For this, we detached cells from the culture dish to measure cell diameter independent on, e.g., process formation. We found a decrease in the cell diameter in N9.ApoKO cells, whereby N9.ApoE4 cells showed no changes (Figure 3A,B). The position and movement of the nucleus inside the cell is a key mechanism for motility and mostly specifies the direction of cell migration (Gomes et al., 2005; Gundersen and Worman, 2013; Bone and Starr, 2016). Cell nuclei are plastic and shaped by intracellular forces, especially the cytoskeleton (Chang et al., 2013; Doyle et al., 2013; Calero-Cuenca et al., 2018). Therefore, we measured likewise the nucleus volume with imaging flow cytometry after DAPI staining. We found an increase in nucleus volume in N9.ApoE4 cells, whereby N9.ApoEKO again showed a decrease (Figure 3C,D).

Taken together, these results displayed a dysregulation in actin cytoskeleton by expression of the human ApoE isoform 4 stressing the importance of ApoE4 as a potential modulator of actin cytoskeleton which determined cell morphology. In contrast, ApoE2 expression cells did not show an effect on cell morphology in our assays.

### ApoE4 Expression Increases Motility of Microglial N9 Cells *in vitro*

**FIGURE 4:**
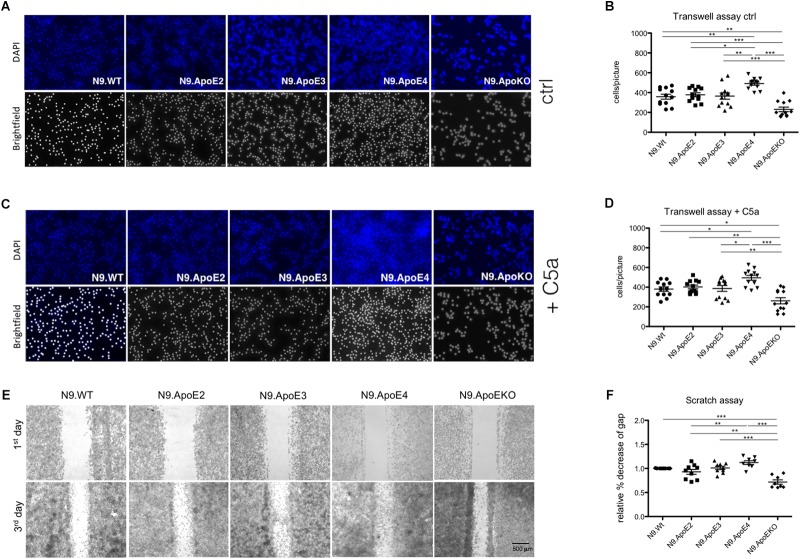
Migration of microglia cells is ApoE-isoform dependent. **(A)** Representative images of motility in transwell assays and **(B)** quantification of migrating cell number demonstrate a significant increase in cell motility of N9.ApoE4 and a significant decrease of N9.ApoEKO (*n* = 12; magnification 10×). **(C,D)** Representative images of migrating cells toward C5a (20 ng/ml) display a significant increase in cell motility of N9.ApoE4 and a significant reduction of N9.ApoEKO migration (*n* = 8; magnification 10×). **(E,F)** Representative images of scratch assay displayed a significant increase in the migration of N9.ApoE4 in comparison to N9.Wt, N9.ApoE2 and N9.ApoEKO confirming the results from transwell assay (*n* = 9). Statistical significance was set up at ^∗^*p* < 0.05, ^∗∗^*p* < 0.01, and ^∗∗∗^*p* < 0.001.

Rearrangement of actin cytoskeleton is a prerequisite for cell migration. Therefore we wanted to asses if expression of N9.ApoE4 likewise changed migration behavior. To determine if ApoE4 specifically influences migration behavior, cell motility was investigated using transwell assay. We observed a significant increase of migration of N9.ApoE4 cells and a significant decrease in N9.ApoEKO in contrast to all other cell lines (Figure 4A,B). Interestingly, ApoE2 expressing cells did not show a change in microglia motility. When we determined migration toward 20 ng/ml of complement factor 5a (C5a) as a chemoattractant we measured a slight increase of cell migration in all five investigated cell lines. Interestingly, the relative differences that we already detected without an additional stimulation persisted. We observed a significant increase of migration in N9.ApoE4 cells and a significant decrease in N9.ApoEKO in contrast to all other cell lines. Again, ApoE2 did not behave different from N9.WT or N9.ApoE3 (Figure 4C,D). Our results therefore rather point to an intrinsic dysregulation of microglia properties in the ApoE4-expressing cells.

In contrast to the directed migration in transwell assay, we also measured general migration using scratch assay. Using this independent method, we confirmed the significant increase in cell motility in N9.ApoE4 in comparison to N9.Wt (Figure 4E,F). Likewise, N9.ApoEKO showed a decrease in directional migration in comparison to the other cell lines. Interestingly, again, we found no influence of the isoform ApoE2 on the migration of N9 cells. To get a more detailed insight into the dysregulation of migration, we assessed expression of *rho-associated coiled-coil-containing protein kinase 1* (*ROCK1*), which is involved in cell motility and actin cytoskeleton rearrangement (Riento and Ridley, 2003). *ROCK1* expression is specifically dysregulated in N9.ApoE4 cells (Supplementary Figure 3) when measured via qPCR and western blot analysis. The intrinsic increase in motility in ApoE4 expressing cells, which could be facilitated by the detected changes in actin cytoskeleton, underlines the putative influence of ApoE-isoforms in microglia physiology. We hypothesize that ApoE4 might lead to an intrinsic dysregulation of microglia without the need of additional stimuli, thus preceding and accelerating the disease-associated phenotype of microglia cells in carriers of ApoE4 in neurodegenerative disease.

### Phagocytosis of Apoptotic Cells Is Increased Whereby Aβ Phagocytosis Is Diminished in ApoE4 Expressing Microglia Cells

**FIGURE 5:**
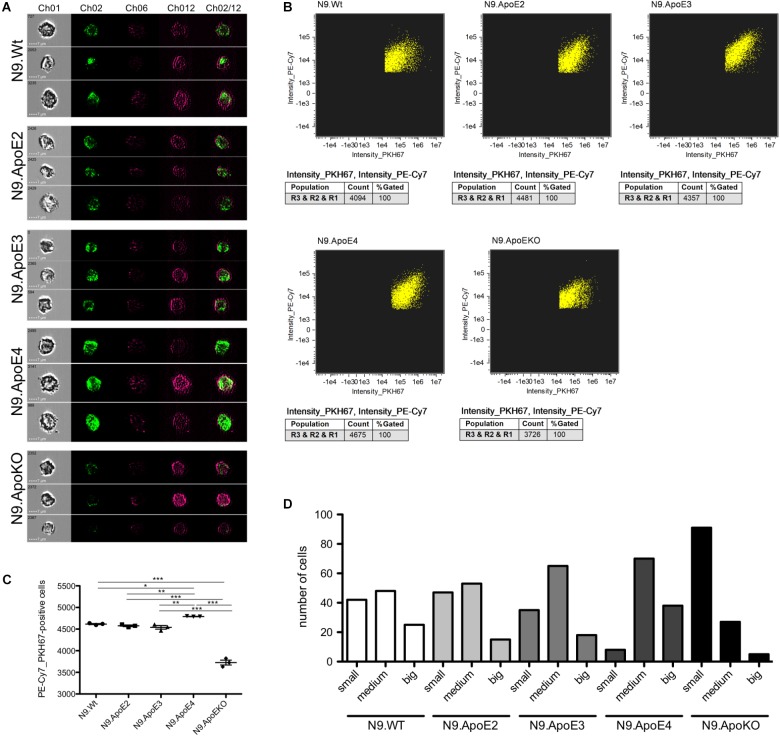
Microglia phagocytosis of apoptotic N2a-cells is upregulated in N9.ApoE4 cells *in vitro*. **(A)** Representative pictures of imaging flow cytometry (Image Stream Mark II, Merk) of N9-cells (purple) after phagocytosis of labeled UV-treated neurons (green). Ch01: brightfield; Ch02: PKH67 (UV-treated N2a cells, apoptotic); Ch06: side scatter; Ch12: PE-Cy7-anti-CD11b (N9-cells); Ch02/12: overlay of Ch02 and Ch12. The images demonstrate the significantly upregulated phagocytosis by N9.ApoE4 in comparison to N9.Wt. Cells with ApoE knockout show a significant decrease in phagocytosing apoptotic cells or debris [a total of 45.000 cells was analyzed in *n* = 3 (including each three technical replicates of 5000 cells)]. **(B)** Gating of PKH67^+^PE-Cy7^+^N9 cells was done with AMARIS software. **(C)** 5.000 N9 cells of each group were quantified with AMARIS and Prism software. **(D)** Quantification of the amount and size of phagocytosed fragments/cells was done manually (small: <∼3.5 μm; medium: ∼3.5–7 μm; big: >∼7 μm). N9.ApoE4 phagocytose more and bigger fragments than N9.Wt and N9.ApoEKO (*n* = 100 cells). Statistical significance was set up at ^∗^*p* < 0.05, ^∗∗^*p* < 0.01, and ^∗∗∗^*p* < 0.001.

Removal of pathogens but also of cellular debris, apoptotic cells, and misfolded proteins from the brain is another key feature of microglia in health and disease (Fu et al., 2014). While in the healthy brain, phagocytosis of synapses and neurons especially during development is mandatory for proper brain function (Stevens and Schafer, 2018) it could be shown that microglia may also cause neuronal death by phagocytosis of stressed but viable neurons (Neher et al., 2011). To investigate the potential influence of ApoE haplotypes specifically on phagocytosis of apoptotic neurons, we determined uptake efficiency of apoptotic cells by our set of N9 microglia expressing the different human ApoE isoforms using imaging flow cytometry. For this, neuronal cells were UV-treated to induce apoptosis and were subsequently fluorescently labeled and then fed to the different N9 microglia cells at a proportion of apoptotic neurons to N9 microglia cells being 1:3. Phagocytosis efficiency of microglia where then measured by imaging flow cytometry and the phagocytosing cells PE-Cy7^+^PKH67^+^ were gated from non-phagocytic PE-Cy7^+^PKH67^-^ cells. Additionally, single cell images of phagocytic microglia were taken. Interestingly, quantification revealed that N9.ApoE4 were significantly more effective in phagocytosis of apoptotic cells than N9.ApoE2 or N9.ApoE3, while N9.ApoEKO showed the lowest uptake efficiency (Figure 5A–C). Next, we analyzed the amount and size of phagocytosed cells and cell fragments in more detail, assessing the content of 100 randomly chosen cells from each group (Figure 5D). Here, we found that N9.ApoEKO phagocytosed preferentially small pieces of labeled material, probably representing cell fragments. In contrast, N9.ApoE4 cells phagocytosed the highest amount of big, round-shaped fragments, presumably representing entire apoptotic cells, in comparison to all other cell lines. Again, ApoE2-expressing cells did not differ from WT or ApoE3-expressing microglia cells (Figure 5A–D).

**FIGURE 6:**
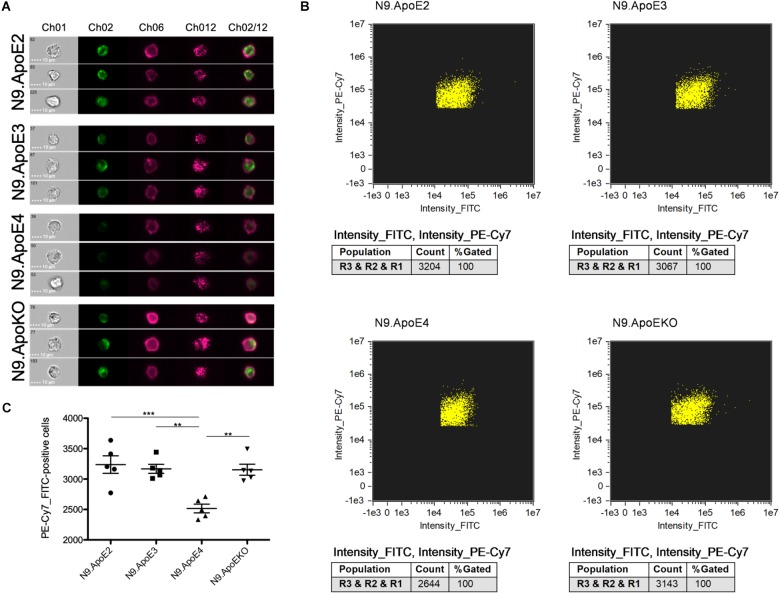
Microglia phagocytosis of aged/oligomerized Aβ_42_ is decreased in N9.ApoE4 cells. **(A)** Representative pictures of imaging flow cytometry of N9 microglia-cells (purple) after phagocytosis of aged/oligomerized FITC-labeled Aβ_42_ (green). Ch01: brightfield; Ch02: FITC (aged/oligomerized Aβ_42_); Ch06: PE-Cy7-anti-CD11b (N9 microglia-cells); Ch12: side scatter; Ch02/12: overlay of Ch02 and Ch06. The images demonstrate the significantly decreased phagocytosis by N9.ApoE4 in comparison to the other cell lines [a total of 75.000 cells was analyzed in *n* = 5 (including each three technical replicates of 5000 cells)]. **(B)** Gating of FITC^+^PE-Cy7^+^N9 cells was done with AMARIS software. **(C)** 5.000 N9 cells of each group were quantified with AMARIS and Prism software. Statistical significance was set up at ^∗∗^*p* < 0.01 and ^∗∗∗^*p* < 0.001.

The increase of phagocytosis efficiency of apoptotic neurons in ApoE4 expressing cells in our model is in striking contrast to the findings that Aβ clearance is rather decreased by the presence of ApoE4 expression. Lin et al. (2018) showed that microglia-like cells differentiate from iPSCs harboring the ApoE4 gene took up Aβ_42_ more slowly than microglia-like cells with the ApoE3 variant. However, the putative beneficial effects of ApoE2 were not assessed in this study. Thus, we wanted to address this feature in our independent model. For this, we set up a phagocytosis assay with aged/oligomerized Aβ_42_ and measured phagocytosing efficiency again via imaging flow cytometry. After incubation of N9 cells with oligomerized/aged Aβ_42_, we observed a significant decrease in the phagocytosis of FITC-labeled Aβ_42_ after 30 min in N9.ApoE4 in comparison to the other cell lines (Figure 6A–C). Contrary to putative beneficial effects of ApoE2 on time of disease onset in familial AD, our N9.ApoE2 cells showed no increase in phagocytosis of the aged/oligomerized Aβ_42_.

Whereby the phagocytosis of apoptotic neurons is increased on the one hand, phagocytosis of aged/oligomerized Aβ_42_ is decreased in microglia cells expressing the ApoE4 isoform. This observations underlines the putative intrinsic dysregulation of ApoE4-expressing microglia toward different cues that they are challenged with in the neurodegenerative brain. Of note, we recently showed *in vivo* that phagocytosis of apoptotic cells *per se* induces a dysregulated, disease like phenotype in microglia (Krasemann et al., 2017). Therefore, the intrinsic ability of ApoE4 to more efficiently phagocytosis of apoptotic cells might start a more severe cycle of dysregulation and synaptic/neuronal toxicity specifically in carriers of the ApoE4 allele. This is in line with recent investigations in a mouse model of tauopathy, where specifically in mice carrying the human ApoE4, widespread neuronal loss could be detected (Shi et al., 2017). Further studies *in vitro* and *in vivo* are warranted to identify if and how the intrinsic dysregulation of ApoE4 microglia might impact selective phagocytosis, microglia dysfunction and neurodegeneration.

## Discussion

ApoE4 is the major genetic risk factor to develop sporadic AD, even if only abundant with one allele (Noguchi et al., 1993; Strittmatter et al., 1993). A variety of models for an ApoE4 specific disease mechanism have been proposed, e.g., several studies have shown a correlation between ApoE4 expression and the increase of Aβ aggregation (Bales et al., 1997). Moreover, it was proposed that the isoform ApoE4 is less anti-inflammatory than the isoform ApoE3 (Barger and Harmon, 1997; Zhu et al., 2012). Interestingly, ApoE4 aggravates neurodegeneration in a mouse model of tauopathy independently of Aβ and renders neurons more vulnerable to degeneration, whereas the deficiency of ApoE is neuroprotective (Shi et al., 2017). In contrast to ApoE4, the isoform ApoE2 was found to act beneficial in familial AD, since it is delaying the age of disease onset (Velez et al., 2016). We could recently show that upregulation of ApoE expression plays a key role in microglia dysregulation in disease (Krasemann et al., 2017). To elucidate, if ApoE isoforms, especially ApoE2 or ApoE4, play a specific role in microglia function in disease, we used the microglial cell line N9 with expression of the different human ApoE isoforms as well as an ApoE knockout. We investigated potential changes in characteristic hallmarks of microglia *in vitro*, since it is difficult to dissect key functions of microglia *in vivo*. We used the microglial cell line N9, which might be superior over BV2 cells or even primary microglia from newborn mice: While N9 show some overlapping expression characteristics with adult microglia, primary microglia isolated from brains of newborn mice display an expression signature that is very different from microglia in the adult brain (Hickman et al., 2008; Butovsky et al., 2014). We overexpressed the three major human ApoE isoforms in N9 cells, which is resembling disease situation and where we likewise detected significant upregulation of ApoE expression in mouse models of neurodegenerative diseases and human AD (Krasemann et al., 2017).

In contrast to Vitek et al. (2009), which showed upregulation of TNFα and IL-6 in microglia derived from newborn APOE4/4 in comparison to APOE3/3 targeted replacement mice, we did not detect ApoE isoform dependent changes of the classical cytokine profile after treatment with LPS. On the other hand, we could detect significant ApoE4 isoform specific influence on the pro-inflammatory and cell-stress associated expression profile after LPS treatment that clearly point to a more disease associated phenotype of the N9.ApoE4 cells. This discrepancy might be due to differences in the model system: While we overexpressed the ApoE isoforms, Vitek et al. (2009) used microglia cells derived from newborn mice which usually show very high expression of ApoE (Butovsky et al., 2014), but that regulate ApoE expression under the endogenous promotor. The latter is targeted by LPS treatment and leads to downregulation of ApoE expression (Supplementary Figure 2C), as we could recently show *in vivo* (Krasemann et al., 2017). Therefore, our stable overexpression may have masked some of the effects that were measured by Vitek et al. (2009), while in our study, the effects that are independent of the ApoE downregulation, but dependent on the ApoE isoform, might have become evident.

In our investigations, we observed changes in cell morphology in an ApoE isoform dependent manner. Vitek et al. (2009) additionally showed via bright field microscopy that microglia derived from APOE4/4 in comparison to APOE3/3 targeted replacement mice demonstrate an altered cell morphology. We likewise detected an altered morphology of ApoE4 expressing cells via confocal microscopy, 3D reconstruction and imaging flow cytometry. In addition to the known data, we demonstrated that N9 cells expressing ApoE4 showed an increase in cell area, cell diameter and nucleus volume, characteristic actin stress fibers as well as an increase in actin expression. Both the formation of actin stress fibers and the nucleus size have been shown to influence actin cytoskeleton as well as cell motility (Byers et al., 1992; Gomes et al., 2005). However, changes in actin dynamics have been shown to be associated with neurological disorders (Pollard and Cooper, 2009). Several studies addressed potential intracellular functions/dysfunctions of ApoE, although the exact mechanisms of these unusual properties were not elucidated to date. Huang and Mahley (2014) reviewed the putative intracellular functions of ApoE including adverse effects on cytoskeletal assembly and stability, mitochondrial integrity and function, and dendritic morphology and function in neurons. They could show that neuronal ApoE, especially ApoE4, is undergoing proteolytic processing which result in C-terminal truncated fragments that cause cytoskeletal changes (Huang and Mahley, 2014). Moreover, it could be shown that intracellular ApoE fragmentation is especially linked to amyloid pathology as in brains of AD mouse models and patients with AD (Huang et al., 2001; Saul and Wirths, 2017). Alternatively, Theendakara et al. (2016) demonstrated that ApoE might also function as a transcription factor in neurons. Interestingly, they reported that ApoE3 and ApoE4 seem to target and influence expression of different genes, with ApoE4 effecting specifically genes associated with microtubule disassembly and synaptic functions (Theendakara et al., 2016). These alternative ApoE functions might also account for the distinct phenotypes that we and others detected in ApoE3- versus ApoE4-expressing microglia. Of note, in our former study, we could show that ApoE is inducing a specific transcriptional pattern in microglia after phagocytosis of apoptotic neurons (Krasemann et al., 2017). However, we did not assess the putative differences of the three human ApoE haplotypes on this regulatory network.

However, ApoE4 may support a more aggressive phenotype in disease situation and might be a factor leading to increased cell motility, as we shown in the results published here. ApoE-dependent migration was also shown by Cudaback et al. (2011) using primary microglia derived from human ApoE targeted replacement mice. While in our model ApoE4 specifically leads to an intrinsically increased migration and ApoE2 has no effect on microglial motility regardless of a stimulus, Cudaback et al. (2011) measured an ApoE isoform-specific modulation of microglial motility only in response to distinct chemotactic stimuli generally associated with neurodegenerative disorders. A putative explanation for the described discrepancy might be the use of different filters for the transwell assays. While we used 8 μm filters as has been utilized by Mazaheri et al. (2017), the Cudaback-study used 10 μm filters. This might have well facilitated mobility toward C5a and led to differences in migration of microglia toward stimuli. Interestingly, using microglia derived from ApoE-KO mice, Cudaback et al. (2011) showed that knockout of ApoE is decreasing migration. A finding that we likewise detected.

Besides migration, phagocytosis is another key function of microglia. Microglia can eliminate pathogens, apoptotic cells, misfolded proteins as well as synapses and processes of living neurons from the brain, which could be beneficial or might be disadvantageous in the context of neurodegenerative diseases (Floden and Combs, 2011; Fu et al., 2014; Hong et al., 2016). Recent studies demonstrate that microglia can cause neuronal death by phagocytosis of stressed but still viable neurons (Brown and Neher, 2014). Of note, Neher et al. (2011) showed that blocking phagocytosis by microglia without affecting inflammation is sufficient for the neurons to survive. Our results showed an upregulation of phagocytosis of apoptotic cells specifically in ApoE4 expressing cells. This might implicate that the isoform ApoE4 starts an unfavorable cycle of phagocytosis and dysregulation. Of note, we have recently shown that phagocytosis of apoptotic neurons induces a specific expression signature in microglia, which is very similar to that seen in disease, including upregulation of ApoE expression (Krasemann et al., 2017). This might be further aggravated by our finding that phagocytosed cells and cell debris was bigger in ApoE4 expressing microglia than that of the other ApoE haplotypes. This is in striking contrast to the uptake of aged/oligomerized Aβ_42_ in our microglia model: Here, we observed a significant decrease in the phagocytosis of aged/oligomerized Aβ_42_ in N9.ApoE4 in comparison to the other cell lines. Our results are in line with data from Lin et al. (2018) showing that ApoE4 expressing microglia-like iPSCs derived cells exhibited a reduced clearance of Aβ compared to those expressing ApoE3. Hu et al. (2015) showed similar effects, but also investigated ApoE2 dependent effects, which resulted in increased Aβ degradation. However, ApoE2 expressing cells displayed no change in phagocytosis of apoptotic cells or Aβ in our model. Li et al. (2012) studied effects of Aβ endocytic trafficking and lysosomal degradation in neurons and could demonstrate a similar ApoE-isoform specific difference with ApoE4^+^-cells being least efficient. In contrast, Pankiewicz et al. (2017) could show that the absolute reduction of Aβ load was greatest in transgenic mice carrying ApoE4 after antibody immunotherapy in aged APP transgenic mice with targeted replacement of murine ApoE with the human ApoE isoforms. This was associated with enhanced co-localization of microglia with Aβ plaques. However, relative reduction levels were not influenced by ApoE genotype. We could recently show that neuronal degeneration in the plaque region in brains of AD model mice is associated with increased expression of ApoE in microglia and onset of microglia dysfunction (Krasemann et al., 2017). It would be interesting to determine if immunotherapies might alternatively stimulate microglia and in this pathway target and decrease ApoE expression levels, thereby partially restoring microglia function, leading to the effects measured in the study by Pankiewicz et al. (2017). However, it has been shown that microglia might well ingest neurons alive under certain conditions (Neher et al., 2011). Such events might be highly important in the context of Aβ pathology, since it has been shown that Aβ seems to render live neurons especially vulnerable to this type of phagocytosis (Neniskyte et al., 2011). Considering our data, it appears that ApoE4 expression specifically predispose microglia toward this detrimental path by upregulating phagocytosis of apoptotic cells and reducing those of Aβ. However, it remains to be clarified whether the current data are transferable to microglia cells *in vivo*.

Our findings implies an intrinsic dysregulation by ApoE4 in different aspects of microglial physiology. The increase in motility and apoptotic-cell phagocytic behavior of N9.ApoE4 microglia cells coupled with decreased phagocytosis of Aβ might lead to an intrinsically higher aggressiveness toward distressed neurons in neurodegeneration. Interestingly, we did not find any influence on microglia cells by the beneficial isoform ApoE2 in our model. However, it is possible that ApoE2 influence other functions of microglia that might not be abundant in the *in vitro* model. Therefore, novel tools for assessing microglia function *in vivo* such as single cell expression analysis should be applied to complement functional studies *in vitro*. Dissecting ApoE isoform dependent microglia functions might help to get novel insights into the details of AD pathology.

## Experimental Procedure

### Cell Culture

N9 microglial cells were cultured in RPMI 1640 (Thermo Fisher Scientific, Waltham, MA, United States) and Neuro2a cells in Dulbecco’s modified Eagle’s medium (DMEM, Thermo Fisher Scientific, Waltham, MA, United States). Both media were supplemented with 10% fetal bovine serum (FBS, GE-Healthcare, Piscataway, NJ, United States). The RPMI media of stable transfected ApoE overexpressing cells (N9.ApoE2, N9.ApoE3, N9.ApoE4) contain Puromycin (2 μg/ml) for selection. Whenever mentioned, the cells were treated for transwell assay with 20 ng/ml of recombinant mouse complement factor C5a (#2150-C5-025, R&D Systems, MN, United States) for 2 h. Additionally, N9 microglia were immune activated to perform subsequent gene expression analysis by stimulation with LPS (Merck, Germany). For this, N9.ApoE3 and N9.ApoE4 cells were treated with 100 ng/ml LPS in serum-free RPMI media for 24 h. Subsequently, RNA was extracted for further analyses.

### Human ApoE Overexpression

Human *ApoE3* (pBabe-PURO containing human *ApoE3*) was purchased from addgene and recloned into pcDNA3.1 (addgene, MA, United States). QuickChange Lightning Site-Directed Mutagenesis Kit (Agilent, CA, United States) was used for mutagenesis to human *ApoE2* (Fwd: 5′-ggtacactgccaggcacttctgcaggtcatc-3′; Rev: 5′-gatgacctgcagaagtgcctggcagtgtacc-3′) and human *ApoE4* (Fwd 5′-caggcggccgcgcacgtcctcca-3′; Rev 5′-tggaggacgtgcgcggccgcctg-3′). These constructs were sequenced and all three confirmed ApoE isoforms were re–cloned and used for lentiviral transduction, which was performed in the “UKE Vector Core Facility.”

### Generation of N9 ApoE Knockout

For *ApoE* knockout in N9 cells we used the Guide-it^TM^ sgRNA *In Vitro* Transcription Kit (Takara Bio, United States) according to the manufacturer’s protocol with the guide RNA 5′-ccggcagcaatgtgaccaacagca-3′. 2.5 × 10^5^ of N9 cells were transfected using the VIROMER Yellow transfection kit (lipocalyx, Halle, Germany) according to the manufacturer’s protocol. Protein and mRNA expression was determined after 48 h. Transfected GFP-positive cells were selected by FACS sorting and plated on a single cell level into wells. 15 Individual clones were further characterized by qPCR and Western Blot analysis.

### Western Blotting

Cells were lysed in Radioimmunoprecipitation assay Buffer (50 mM Tris pH 7.2, 150 mM NaCl, 0.1% sodium dodecyl sulfate (SDS), 0.5% sodium deoxycholate, 1% Triton X100, 1% EDTA (0.5 M), 1% NP40) containing protease inhibitor cocktail (Roche). Protein concentration of lysates were determined by Bradford assay (Bio-Rad Laboratories, Hercules, CA, United States). Lysate was mixed with sample buffer (0.5 M Tris–HCL, pH 6.8, 15% SDS, 50% glycerol, 25% Aβ-mercaptoethanol, 0.01% bromophenol blue), incubated at 95°C for 5 min and loaded onto a 12 or 8% Bis/Tris SDS gel. Proteins were detected with different primary antibodies in a Li-COR Odyssey CLx system and signal strength were quantified with Image Studio Version 5.2 (LI-COR, Nebraska, United States).

Sources of the antibodies were as follows: anti-ApoE (Merck, AB947), anti-PDI (StressMarq, SPC-114). Fluorescence-conjugated secondary antibodies were purchased from Invitrogen, Thermo Scientific as followed: donkey-anti-mouse (Alexa 790, A11371), goat-anti-mouse (Alexa 680, A28183), goat-anti-rabbit (Alexa 680, A21109), goat-anti-rabbit (Alexa 790, A27041), rabbit-anti-goat (Alexa 680, A27020).

### Imaging Flow Cytometry (Image Stream X Mark II, Merck)

For investigation of the nucleus size, 5 × 10^6^ N9 cells were detached from the plate, fixated in 4% PFA for 20 min and filtrated (30 μm). N9 cells were stained for 5 min with DAPI (1 μg/ml) and diluted into Imaging Buffer. Per experiment, we measured 5.000 N9 cells (magnification: 40×, fluidics: high). Quantification was done by gating for DAPI-positive nuclei. Afterward volume of the nucleus was calculated using a specific mask from IDEAS 6.2 software.

For phagocytosis assay 5 × 10^5^ N9 cells were seeded a day before the experiment. In parallel, 2 × 10^5^ N2a neuronal cells were seeded and apoptosis was induced after 24 h in N2a cells by treatment with UV-light for 30 min and subsequent 2 h of incubation at 37°C and 5% CO_2_. Afterward, neuronal N2a cells were stained with PKH67 (1:250) (Merck, Germany) for 5 min, blocked with FBS (1:10) and washed thoroughly with PBS. 3 × 10^5^ stained, apoptotic N2a were fed to the plated N9 cells and incubated overnight. Next day, N9 cells were washed to remove remaining N2a cells, detached from the plate, fixated with 4% PFA for 20 min and filtrated (30 μm). N9 cells were stained for 20 min with PE-Cy7-CD11b antibody (1:50, anti-CD11b monoclonal antibody (M1/70), PE-Cyanine 7, eBioscience, #25-0112-82, Thermo Fisher Scientific, CA, United States) and diluted into Imaging Buffer (PBS, 1% EDTA, 2% FBS). Per experiment and ApoE-isotype, we measured 5.000 PE-Cy7-positive N9 cells (magnification: 40×, fluidics: high). Quantification was done by gating for PE-Cy7- and PKH67-positive cells (phagocytic cells) and comparison to total measured cells.

For investigation of microglial phagocytosis of FITC-labeled Aβ_42_ we first prepared the aged/oligomerized Aβ_42_. 1 mg/ml stock solution of Aβ_42_ (GenicBio, Shanghai, China) was prepared by dissolving the lyophilized powder in 1 ml 1× PBS containing 0.1% NaOH. The solution was sonicated for 15 min at room temperature in a water bath, then incubated on a shaker at 37°C under heavy agitation for 1 h and incubated at room temperature for at least 24 h. N9 cells were incubated with aged/oligomerized Aβ_42_ (1 μg/ml) for 30 min. After incubation, the N9 cells were intensively washed to remove excess Aβ and prepared for imaging flow cytometry as described before.

### Transwell Migration Assay

Directed migration through a membrane toward media or 20 ng/ml of recombinant mouse complement component C5a (#2150-C5-025, R&D Systems, MN, United States) was investigated in transwell assays (COSTAR 24 well plate with inserts, 8 μm pore, Corning, NY, United States). 5 × 10^5^ N9 cells were seeded in 100 μl media (RPMI complemented with GlutaMAX^TM^, Thermo Fisher Scientific, Waltham, MA, United States) in the top well while media alone or C5a containing media (600 μl) was added to the bottom well. Cells were incubated for 2 h at 37°C and 5% CO_2_. After incubation time the stationary cells were removed from the top. Cells which migrated through the filter were fixed with 4% PFA, followed by permeabilization with 0.2% TritonX for 10 min (Roche, Germany). After cell staining with DAPI (1 μg/ml), 6 images of representative regions of each transwell were taken with an ApoTOME (Zeiss) and migrated cells were quantified using Fiji software with “cell counter” plugin.

### Scratch Assay

Overall migration of N9 was assayed with 2 well migration inserts (Culture-Insert 2 Well in μm dish 35 mm, ibidi, #81176, Germany). 5 × 10^4^ N9 cells were seeded into each insert-chambers in 70 μl media (RPMI, Thermo Fisher Scientific, Waltham, MA, United States). After 24 h, inserts were removed and 1 ml of media was added to the dish (Thermo Fisher Scientific; Waltham, MA, United States). Documentation was performed every day by taking six representative pictures with an ApoTOME (Zeiss) until the migrating cells covered the scratch. Quantification was done with Fiji plugin “MRI wound healing tool” with the first and the last day of assay documentation.

### RNA Expression Analyses

Total RNA was isolated using the NucleoSpin RNA purification kit (#740955.250, Macherey-Nagel, Germany) according to the manufacturer’s protocol and cDNA was generated from 500 ng isolated RNA with TaqMan reverse transcription polymerase chain reaction (TaqMan^TM^ Reverse Transcription Reagents, #N8080234, Thermo Fisher Scientific, Waltham, MA, United States). mRNA expression levels were determined using EXPRESS qPCR Supermix (#1178501K, Thermo Fisher Scientific, Waltham, MA, United States) and TaqMan gene expression assays (Cat.No #4331182; Assay ID: Mm01307193_g1; Hs00171168; Mm99999915) in a QuantStudio 5 (Thermo Fisher Scientific, Waltham, MA, United States) with the following standard program: 95°C for 10 min, 40 cycles: 95°C for 15 s, 60°C for 1 min. qPCRs were performed in triplicates for each sample (*n* = 1 correspond to 3 replicates). Data sets were normalized relative to *Gapdh* and are presented as relative expression in comparison to *Gapdh* as mean +/- s.e.m.

Alternatively, multiplexed target profiling was performed using the “Mouse Neuroinflammation expression panel” with the NanoString nCounter technology^1^. 50 ng of total RNA per sample were used in the nCounter panel to directly measure RNA-counts according to the manufacturer’s suggested protocol. NanoString data were normalized and analyzed using nSolver 4.0 software. RNA counts were normalized using the geometric mean of the housekeeping genes included in the panel, after validation against positive and negative controls. Background thresholding was fixed at less than 20 counts. Fold changes were calculated using the average of each group and were normalized. Fold changes were calculated comparing treated and untreated cells or comparing between ApoE3 and Apo4 haplotypes. *T*-tests were calculated for pair-wise analyses followed by false discovery rate adjusted *p*-value calculation using the Benjamini–Yekutieli method. Heatmaps and clustering were generated in R (version 3.3.2) using heatmap.2 from the gplots package. Volcano plots were generated by nSolver advance analysis 2.0 package.

### Immunofluorescent Microscopy and Quantification

N9 were seeded in channel slides (μ-slide VI 0.5 glass bottom, ibidi, Germany) at a density of 4 × 10^3^ cells per channel. Cells were fixed after 24 h with 4% PFA, followed by permeabilization with 0.2% of TritonX (Roche, Germany) and blocked with Blocking Buffer (Pierce Protein-Free T20 (TBS) Blocking Buffer, Thermo Fisher Scientific, United States). Primary antibody (Goat anti-Apolipoprotein E, 1:500, #AB947, Merck, Germany) was incubated overnight at 4°C. After washing, secondary antibody (Rabbit anti-goat IgG Superclonal, Alexa Fluor 680, 1:50, Thermo Fisher Scientific) was incubated for 90 min. After repeated washing, actin was stained with Rhodamine-Phalloidin (Thermo Fisher Scientific) for 20 min at RT. Finally, cells were mounted with 4.6-diamidino-2-phenylindole (DAPI) Fluoromount-G (Southern Biotech, Birmingham, AL, United States) and images were taken with a TCS SP8 (Leica Microsystems, Wetzlar, Germany) confocal microscope. Analysis was done with Fiji and IMARIS ×64 software.

### Statistically Analysis of Data

Statistically analyses were performed using GraphPad Prism 5 (LaJolla, CA, United States). Experimental groups were compared using students-*t*-test and one-way-ANOVA (followed by Tukey’s *post hoc* test). Statistical significance was set up at *p*-values < 0.05 (^∗^), < 0.01 (^∗∗^), < 0.001 (^∗∗∗^), < 0.0001 (^∗∗∗∗^). Data were plotted using GraphPad Prism 5 showing standard error of the mean.

## Author Contributions

SK and CM conceived the idea, designed the experiments, and coordinated the study. CM performed all experiments with support of AH, analyzed the data. DS-F analyzed the expression of N9 cells after LPS treatment. CM and SK wrote the manuscript with input of MG and DS-F. All authors approved the final version of the manuscript.

## Conflict of Interest Statement

The authors declare that the research was conducted in the absence of any commercial or financial relationships that could be construed as a potential conflict of interest.

## Footnotes

1 http://www.nanostring.com/

## Supplementary Material

FIGURE S1 Actin expression is increased in N9.ApoE4 cells. **(A)** qPCR analysis of murine *ApoE* RNA-expression in transfected cells normalized against murine *GapDH* levels. All cell lines except N9.ApoEKO express murine *ApoE* at a very low, endogenous level. **(B)** Actin expression of N9.ApoE4 was significant increase in microglia cells when investigated using confocal microscopy in comparison to N9.Wt (*n* = 5). **(C)** The significant increase in *actin* expression of N9.ApoE4 cells could be confirmed by qPCR (normalized against *Gapdh*, *n* = 5).

FIGURE S2 Proinflammatory signaling is altered in N9.ApoE4 after LPS treatment. The NanoString nCounter Mouse Neuroinflammation Panel was used to evaluate gene expression analysis of inflammation-relevant genes, in N9.ApoE3 and N9.ApoE4 after 24 h of treatment with 100 ng/ml LPS. **(A)** Heatmap depicting cluster analysis for gene expression levels of 389 out of the 770 genes included in the panel that presented differential expression (upregulated red, downregulated blue) between N9.ApoE3 cells as controls (*n* = 3, black bar) and LPS treated ApoE3 (*n* = 3 blue bar) and ApoE4 (*n* = 3, red bar) N9 cells. **(B)** Volcano plot for *t*-test pairwise analysis between LPS treated and control N9.ApoE3 cells. 152 genes showed to be differentially regulated between treated N9.ApoE3 cells and controls after adjusting for false discovery rate (FDR) using the Benjamini–Yekutieli procedure (see Supplementary Table 1). **(C)** Boxplots displaying significantly different expression in LPS treated and untreated ApoE3/4 N9 cells after Two-way ANOVA analysis. Expression levels of Bruton tyrosine kinase (Btk), Myocyte Enhancer Factor 2C (MEF2c) and Triggering receptor expressed on myeloid cells 2 (Trem2) are significantly decreased in LPS treated cells, particularly in N9.ApoE4 treated cells. Conversely, expression levels of Interleukin-1 receptor antagonist (Il1rn), Sequestosome-1 (Sqstm1) and TNF receptor-associated factor 2 (Traf2), showed to be significantly upregulated after LPS treatment, with higher expression levels in N9.ApoE4 treated cells. This trend was not observed in Transcription factor MafF (Maff) expression levels, in which, although significantly upregulated in ApoE transfected N9 cells after LPS treatment, N9.ApoE4 cells expressed significantly lower levels than N9.ApoE3 cells. ApoE expression levels of the endogenous mouse ApoE are depicted as controls, with LPS treated cells showing significantly lower levels, (*n* = 3, see also Supplementary Table 2). **(D)** Volcano plot for *t*-test pairwise analysis between LPS treated N9.ApoE3 versus N9.ApoE4 cells. Nine genes showed to be differentially regulated between treated cells after adjusting for FDR using the Benjamini–Yekutieli procedure (see Supplementary Table 3).

FIGURE S3 ROCK1 expression is dysregulated in N9.ApoE4. **(A)** Western Blot analysis of ROCK1. PDI displays protein loading. **(B)** Quantification shows a significant downregulation of ROCK1 in N9.ApoE4 in comparison to N9.Wt (*n* = 9). **(C)** Quantification of RNA-expression level of *Rock1* shows a significant decrease in N9.ApoE4 in comparison to N9.Wt (*n* = 7). Murine *GapDH* were used as endogenous control.

TABLE S1 Pairwise analysis of gene expression levels between untreated N9.ApoE3, and LPS treated N9.ApoE3 cells.

TABLE S2 Two-way ANOVA analysis of gene expression levels between untreated N9.ApoE3, LPS treated N9.ApoE3, and LPS treated N9.ApoE4 cells.

TABLE S3 Pairwise analysis of gene expression levels between LPS treated N9.ApoE3, and N9.ApoE4 cells.

## References

[B1] Bales K. R. Verina T. Dodel R. C. Du Y. Altstiel L. Bender M. (1997). Lack of apolipoprotein E dramatically reduces amyloid beta-peptide deposition. *Nat. Genet.* 17 263–264. 10.1038/ng1197-263 9354781

[B2] Barger S. W. Harmon A. D. (1997). Microglial activation by Alzheimer amyloid precursor protein and modulation by apolipoprotein E. *Nature* 388 878–881. 10.1038/42257 9278049

[B3] Brown G. C. Neher J. J. (2014). Microglial phagocytosis of live neurons. *Nat. Rev. Neurosci.* 15 209–216. 10.1038/nrn3710 24646669

[B4] Bone C. R. Starr D. A. (2016). Nuclear migration events throughout development. *J. Cell Sci.* 129 1951–1961. 10.1242/jcs.179788 27182060 PMC5998656

[B5] Bu G. (2009). Apolipoprotein E and its receptors in Alzheimer’s disease: pathways, pathogenesis and therapy. *Nat. Rev. Neurosci.* 10 333–344. 10.1038/nrn2620 19339974 PMC2908393

[B6] Butovsky O. Jedrychowski M. P. Cialic R. Krasemann S. Murugaiyan G. Fanek Z. (2015). Targeting miR-155 restores abnormal microglia and attenuates disease in SOD1 mice. *Ann. Neurol.* 77 75–99. 10.1002/ana.24304 25381879 PMC4432483

[B7] Butovsky O. Jedrychowski M. P. Moore C. S. Cialic R. Lanser A. J. Gabriely G. (2014). Identification of a unique TGF-beta-dependent molecular and functional signature in microglia. *Nat. Neurosci.* 17 131–143. 10.1038/nn.3599 24316888 PMC4066672

[B8] Byers H. R. Etoh T. Vink J. Franklin N. Gattoni-Celli S. Mihm M. C. Jr. (1992). Actin organization and cell migration of melanoma cells relate to differential expression of integrins and actin-associated proteins. *J. Dermatol.* 19 847–852. 10.1111/j.1346-8138.1992.tb03795.x 1293173

[B9] Calero-Cuenca F. J. Janota C. S. Gomes E. R. (2018). Dealing with the nucleus during cell migration. *Curr. Opin. Cell Biol.* 50 35–41. 10.1016/j.ceb.2018.01.014 29454272

[B10] Chang W. Folker E. S. Worman H. J. Gundersen G. G. (2013). Emerin organizes actin flow for nuclear movement and centrosome orientation in migrating fibroblasts. *Mol. Biol. Cell* 24 3869–3880. 10.1091/mbc.E13-06-0307 24152738 PMC3861083

[B11] Chiu I. M. Morimoto E. T. Goodarzi H. Liao J. T. O’keeffe S. Phatnani H. P. (2013). A neurodegeneration-specific gene-expression signature of acutely isolated microglia from an amyotrophic lateral sclerosis mouse model. *Cell Rep.* 4 385–401. 10.1016/j.celrep.2013.06.018 23850290 PMC4272581

[B12] Corder E. H. Saunders A. M. Strittmatter W. J. Schmechel D. E. Gaskell P. C. Small G. W. (1993). Gene dose of apolipoprotein E type 4 allele and the risk of Alzheimer’s disease in late onset families. *Science* 261 921–923. 10.1126/science.8346443 8346443

[B13] Cudaback E. Li X. Montine K. S. Montine T. J. Keene C. D. (2011). Apolipoprotein E isoform-dependent microglia migration. *FASEB J.* 25 2082–2091. 10.1096/fj.10-176891 21385991 PMC3101033

[B14] Dibaj P. Nadrigny F. Steffens H. Scheller A. Hirrlinger J. Schomburg E. D. (2010). NO mediates microglial response to acute spinal cord injury under ATP control in vivo. *Glia* 58 1133–1144. 10.1002/glia.20993 20468054

[B15] Doyle A. D. Petrie R. J. Kutys M. L. Yamada K. M. (2013). Dimensions in cell migration. *Curr. Opin. Cell Biol.* 25 642–649. 10.1016/j.ceb.2013.06.004 23850350 PMC3758466

[B16] Floden A. M. Combs C. K. (2011). Microglia demonstrate age-dependent interaction with amyloid-beta fibrils. *J. Alzheimers Dis.* 25 279–293. 10.3233/JAD-2011-101014 21403390 PMC3295838

[B17] Fu R. Shen Q. Xu P. Luo J. J. Tang Y. (2014). Phagocytosis of microglia in the central nervous system diseases. *Mol. Neurobiol.* 49 1422–1434. 10.1007/s12035-013-8620-6 24395130 PMC4012154

[B18] Gautier E. L. Shay T. Miller J. Greter M. Jakubzick C. Ivanov S. (2012). Gene-expression profiles and transcriptional regulatory pathways that underlie the identity and diversity of mouse tissue macrophages. *Nat. Immunol.* 13 1118–1128. 10.1038/ni.2419 23023392 PMC3558276

[B19] Gomes E. R. Jani S. Gundersen G. G. (2005). Nuclear movement regulated by Cdc42, MRCK, myosin, and actin flow establishes MTOC polarization in migrating cells. *Cell* 121 451–463. 10.1016/j.cell.2005.02.022 15882626

[B20] Gundersen G. G. Worman H. J. (2013). Nuclear positioning. *Cell* 152 1376–1389. 10.1016/j.cell.2013.02.031 23498944 PMC3626264

[B21] Heneka M. T. Carson M. J. El Khoury J. Landreth G. E. Brosseron F. Feinstein D. L. (2015). Neuroinflammation in Alzheimer’s disease. *Lancet Neurol.* 14 388–405. 10.1016/S1474-4422(15)70016-5 25792098 PMC5909703

[B22] Hickman S. E. Allison E. K. El Khoury J. (2008). Microglial dysfunction and defective beta-amyloid clearance pathways in aging Alzheimer’s disease mice. *J. Neurosci.* 28 8354–8360. 10.1523/JNEUROSCI.0616-08.2008 18701698 PMC2597474

[B23] Hickman S. E. Kingery N. D. Ohsumi T. K. Borowsky M. L. Wang L. C. Means T. K. (2013). The microglial sensome revealed by direct RNA sequencing. *Nat. Neurosci.* 16 1896–1905. 10.1038/nn.3554 24162652 PMC3840123

[B24] Holtman I. R. Raj D. D. Miller J. A. Schaafsma W. Yin Z. Brouwer N. (2015). Induction of a common microglia gene expression signature by aging and neurodegenerative conditions: a co-expression meta-analysis. *Acta Neuropathol. Commun.* 3:31. 10.1186/s40478-015-0203-5 26001565 PMC4489356

[B25] Hong S. Beja-Glasser V. F. Nfonoyim B. M. Frouin A. Li S. Ramakrishnan S. (2016). Complement and microglia mediate early synapse loss in Alzheimer mouse models. *Science* 352 712–716. 10.1126/science.aad8373 27033548 PMC5094372

[B26] Hu J. Liu C. C. Chen X. F. Zhang Y. W. Xu H. Bu G. (2015). Opposing effects of viral mediated brain expression of apolipoprotein E2 (apoE2) and apoE4 on apoE lipidation and Abeta metabolism in apoE4-targeted replacement mice. *Mol. Neurodegener.* 10:6. 10.1186/s13024-015-0001-3 25871773 PMC4356137

[B27] Huang Y. Liu X. Q. Wyss-Coray T. Brecht W. J. Sanan D. A. Mahley R. W. (2001). Apolipoprotein E fragments present in Alzheimer’s disease brains induce neurofibrillary tangle-like intracellular inclusions in neurons. *Proc. Natl. Acad. Sci. U.S.A.* 98 8838–8843. 10.1073/pnas.151254698 11447277 PMC37522

[B28] Huang Y. Mahley R. W. (2014). Apolipoprotein E: structure and function in lipid metabolism, neurobiology, and Alzheimer’s diseases. *Neurobiol. Dis.* 72(Pt A), 3–12. 10.1016/j.nbd.2014.08.025 25173806 PMC4253862

[B29] Huynh T. V. Davis A. A. Ulrich J. D. Holtzman D. M. (2017). Apolipoprotein E and Alzheimer’s disease: the influence of apolipoprotein E on amyloid-beta and other amyloidogenic proteins. *J. Lipid Res.* 58 824–836. 10.1194/jlr.R075481 28246336 PMC5408619

[B30] Jucker M. Walker L. C. (2011). Pathogenic protein seeding in Alzheimer disease and other neurodegenerative disorders. *Ann. Neurol.* 70 532–540. 10.1002/ana.22615 22028219 PMC3203752

[B31] Keren-Shaul H. Spinrad A. Weiner A. Matcovitch-Natan O. Dvir-Szternfeld R. Ulland T. K. (2017). A unique microglia type associated with restricting development of Alzheimer’s disease. *Cell* 169 1276–1290.e17. 10.1016/j.cell.2017.05.018 28602351

[B32] Kettenmann H. Hanisch U. K. Noda M. Verkhratsky A. (2011). Physiology of microglia. *Physiol. Rev.* 91 461–553. 10.1152/physrev.00011.2010 21527731

[B33] Krasemann S. Madore C. Cialic R. Baufeld C. Calcagno N. El Fatimy R. (2017). The TREM2-APOE pathway drives the transcriptional phenotype of dysfunctional microglia in neurodegenerative diseases. *Immunity* 47 566–581.e9. 10.1016/j.immuni.2017.08.008 28930663 PMC5719893

[B34] Kreutzberg G. W. (1996). Microglia: a sensor for pathological events in the CNS. *Trends Neurosci.* 19 312–318. 10.1016/0166-2236(96)10049-7 8843599

[B35] Leu T. H. Maa M. C. (2003). Functional implication of the interaction between EGF receptor and c-Src. *Front. Biosci.* 8:s28–s38. 10.2741/980 12456372

[B36] Li J. Kanekiyo T. Shinohara M. Zhang Y. Ladu M. J. Xu H. (2012). Differential regulation of amyloid-beta endocytic trafficking and lysosomal degradation by apolipoprotein E isoforms. *J. Biol. Chem.* 287 44593–44601. 10.1074/jbc.M112.420224 23132858 PMC3531774

[B37] Lin Y. T. Seo J. Gao F. Feldman H. M. Wen H. L. Penney J. (2018). APOE4 causes widespread molecular and cellular alterations associated with alzheimer’s disease phenotypes in human iPSC-derived brain cell types. *Neuron* 98:1294. 10.1016/j.neuron.2018.06.011 29953873 PMC6048952

[B38] Liu C. C. Liu C. C. Kanekiyo T. Xu H. Bu G. (2013). Apolipoprotein E and Alzheimer disease: risk, mechanisms and therapy. *Nat. Rev. Neurol.* 9 106–118. 10.1038/nrneurol.2012.263 23296339 PMC3726719

[B39] Mazaheri F. Snaidero N. Kleinberger G. Madore C. Daria A. Werner G. (2017). TREM2 deficiency impairs chemotaxis and microglial responses to neuronal injury. *EMBO Rep.* 18 1186–1198. 10.15252/embr.201743922 28483841 PMC5494532

[B40] Neher J. J. Neniskyte U. Zhao J. W. Bal-Price A. Tolkovsky A. M. Brown G. C. (2011). Inhibition of microglial phagocytosis is sufficient to prevent inflammatory neuronal death. *J. Immunol.* 186 4973–4983. 10.4049/jimmunol.1003600 21402900

[B41] Neniskyte U. Neher J. J. Brown G. C. (2011). Neuronal death induced by nanomolar amyloid beta is mediated by primary phagocytosis of neurons by microglia. *J. Biol. Chem.* 286 39904–39913. 10.1074/jbc.M111.267583 21903584 PMC3220594

[B42] Nimmerjahn A. Kirchhoff F. Helmchen F. (2005). Resting microglial cells are highly dynamic surveillants of brain parenchyma in vivo. *Science* 308 1314–1318. 10.1126/science.1110647 15831717

[B43] Noguchi S. Murakami K. Yamada N. (1993). Apolipoprotein E genotype and Alzheimer’s disease. *Lancet* 342:737. 10.1016/0140-6736(93)91728-5 8103832

[B44] Olah M. Patrick E. Villani A. C. Xu J. White C. C. Ryan K. J. (2018). A transcriptomic atlas of aged human microglia. *Nat. Commun.* 9:539. 10.1038/s41467-018-02926-5 29416036 PMC5803269

[B45] Orre M. Kamphuis W. Osborn L. M. Melief J. Kooijman L. Huitinga I. (2014). Acute isolation and transcriptome characterization of cortical astrocytes and microglia from young and aged mice. *Neurobiol. Aging* 35 1–14. 10.1016/j.neurobiolaging.2013.07.008 23954174

[B46] Pankiewicz J. E. Baquero-Buitrago J. Sanchez S. Lopez-Contreras J. Kim J. Sullivan P. M. (2017). APOE genotype differentially modulates effects of anti-abeta, passive immunization in APP transgenic mice. *Mol. Neurodegener.* 12:12. 10.1186/s13024-017-0156-1 28143566 PMC5282859

[B47] Pantaloni D. Le Clainche C. Carlier M. F. (2001). Mechanism of actin-based motility. *Science* 292 1502–1506. 10.1126/science.1059975 11379633

[B48] Pitas R. E. Boyles J. K. Lee S. H. Foss D. Mahley R. W. (1987). Astrocytes synthesize apolipoprotein E and metabolize apolipoprotein E-containing lipoproteins. *Biochim. Biophys. Acta* 917 148–161. 10.1016/0005-2760(87)90295-5 3539206

[B49] Pollard T. D. Cooper J. A. (2009). Actin, a central player in cell shape and movement. *Science* 326 1208–1212. 10.1126/science.1175862 19965462 PMC3677050

[B50] Ransohoff R. M. Perry V. H. (2009). Microglial physiology: unique stimuli, specialized responses. *Annu. Rev. Immunol.* 27 119–145. 10.1146/annurev.immunol.021908.132528 19302036

[B51] Rebeck G. W. Reiter J. S. Strickland D. K. Hyman B. T. (1993). Apolipoprotein E in sporadic Alzheimer’s disease: allelic variation and receptor interactions. *Neuron* 11 575–580. 10.1016/0896-6273(93)90070-8 8398148

[B52] Reiman E. M. Chen K. Liu X. Bandy D. Yu M. Lee W. (2009). Fibrillar amyloid-beta burden in cognitively normal people at 3 levels of genetic risk for Alzheimer’s disease. *Proc. Natl. Acad. Sci. U.S.A.* 106 6820–6825. 10.1073/pnas.0900345106 19346482 PMC2665196

[B53] Riento K. Ridley A. J. (2003). Rocks: multifunctional kinases in cell behaviour. *Nat. Rev. Mol. Cell Biol.* 4 446–456. 10.1038/nrm1128 12778124

[B54] Roses A. D. (1996). Apolipoprotein E alleles as risk factors in Alzheimer’s disease. *Annu. Rev. Med.* 47 387–400. 10.1146/annurev.med.47.1.387 8712790

[B55] Saul A. Wirths O. (2017). Endogenous apolipoprotein E (ApoE) fragmentation is linked to amyloid pathology in transgenic mouse models of Alzheimer’s disease. *Mol. Neurobiol.* 54 319–327. 10.1007/s12035-015-9674-4 26742512

[B56] Schmechel D. E. Saunders A. M. Strittmatter W. J. Crain B. J. Hulette C. M. Joo S. H. (1993). Increased amyloid beta-peptide deposition in cerebral cortex as a consequence of apolipoprotein E genotype in late-onset Alzheimer disease. *Proc. Natl. Acad. Sci. U.S.A.* 90 9649–9653. 10.1073/pnas.90.20.9649 8415756 PMC47627

[B57] Shi Y. Yamada K. Liddelow S. A. Smith S. T. Zhao L. Luo W. (2017). ApoE4 markedly exacerbates tau-mediated neurodegeneration in a mouse model of tauopathy. *Nature* 549 523–527. 10.1038/nature24016 28959956 PMC5641217

[B58] Stevens B. Schafer D. P. (2018). Roles of microglia in nervous system development, plasticity, and disease. *Dev. Neurobiol.* 78 559–560. 10.1002/dneu.22594 29704318

[B59] Strittmatter W. J. Saunders A. M. Schmechel D. Pericak-Vance M. Enghild J. Salvesen G. S. (1993). Apolipoprotein E: high-avidity binding to beta-amyloid and increased frequency of type 4 allele in late-onset familial Alzheimer disease. *Proc. Natl. Acad. Sci. U.S.A.* 90 1977–1981. 10.1073/pnas.90.5.1977 8446617 PMC46003

[B60] Tamboli I. Y. Heo D. Rebeck G. W. (2014). Extracellular proteolysis of apolipoprotein E (apoE) by secreted serine neuronal protease. *PLoS One* 9:e93120. 10.1371/journal.pone.0093120 24675880 PMC3968057

[B61] Theendakara V. Peters-Libeu C. A. Spilman P. Poksay K. S. Bredesen D. E. Rao R. V. (2016). Direct transcriptional effects of apolipoprotein E. *J. Neurosci.* 36 685–700. 10.1523/JNEUROSCI.3562-15.2016 26791201 PMC4719010

[B62] Tojkander S. Gateva G. Lappalainen P. (2012). Actin stress fibers–assembly, dynamics and biological roles. *J. Cell Sci.* 125 1855–1864. 10.1242/jcs.098087 22544950

[B63] Uchihara T. Duyckaerts C. He Y. Kobayashi K. Seilhean D. Amouyel P. (1995). ApoE immunoreactivity and microglial cells in Alzheimer’s disease brain. *Neurosci. Lett.* 195 5–8. 10.1016/0304-3940(95)11763-m 7478253

[B64] Velez J. I. Lopera F. Sepulveda-Falla D. Patel H. R. Johar A. S. Chuah A. (2016). APOE^∗^E2 allele delays age of onset in PSEN1 E280A Alzheimer’s disease. *Mol. Psychiatry* 21 916–924. 10.1038/mp.2015.177 26619808 PMC5414071

[B65] Vitek M. P. Brown C. M. Colton C. A. (2009). APOE genotype-specific differences in the innate immune response. *Neurobiol. Aging* 30 1350–1360. 10.1016/j.neurobiolaging.2007.11.014 18155324 PMC2782461

[B66] Wehrle-Haller B. Imhof B. A. (2003). Actin, microtubules and focal adhesion dynamics during cell migration. *Int. J. Biochem. Cell Biol.* 35 39–50. 10.1016/s1357-2725(02)00071-7 12467646

[B67] Yu J. T. Tan L. Hardy J. (2014). Apolipoprotein E in Alzheimer’s disease: an update. *Annu. Rev. Neurosci.* 37 79–100. 10.1146/annurev-neuro-071013-014300 24821312

[B68] Zhu Y. Nwabuisi-Heath E. Dumanis S. B. Tai L. M. Yu C. Rebeck G. W. (2012). APOE genotype alters glial activation and loss of synaptic markers in mice. *Glia* 60 559–569. 10.1002/glia.22289 22228589 PMC3276698

