# Supplementary figures and images for: Phagocytosis of Apoptotic Cells Is Specifically Upregulated in ApoE4 Expressing Microglia in vitro
Source: Front Cell Neurosci. 2019 May 3;13:181. doi: 10.3389/fncel.2019.00181 (PMC6509203; doi:10.3389/fncel.2019.00181)

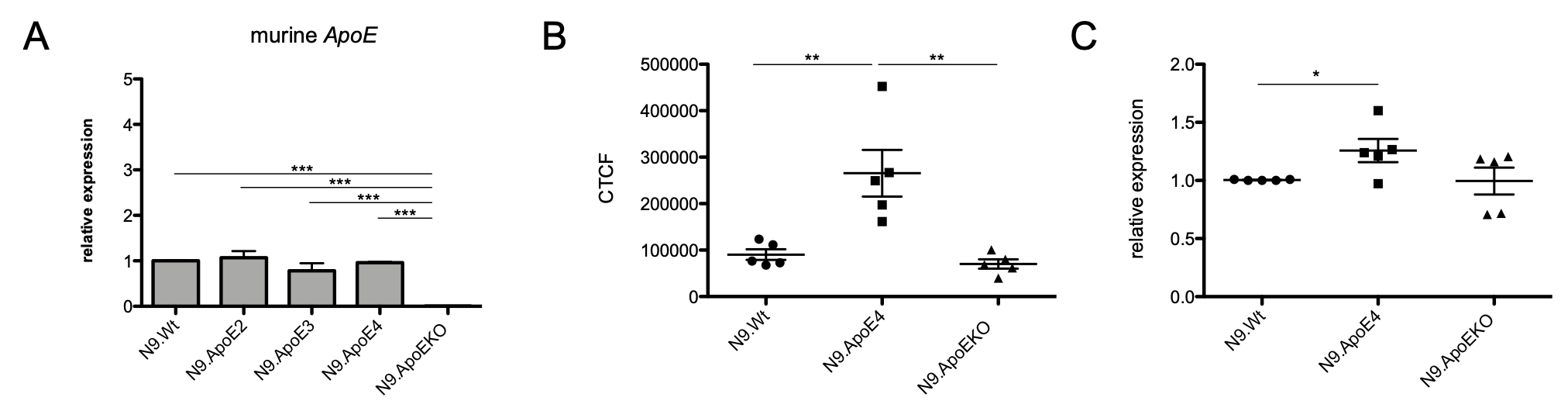

Supplement: FIGURE S1 — Actin expression is increased in N9.ApoE4 cells. (A) qPCR analysis of murine ApoE RNA-expression in transfected cells normalized against murine GapDH levels. All cell lines except N9.ApoEKO express murine ApoE at a very low, endogenous level. (B) Actin expression of N9.ApoE4 was significant increase in microglia cells when investigated using confocal microscopy in comparison to N9.Wt (n = 5). (C) The significant increase in actin expression of N9.ApoE4 cells could be confirmed by qPCR (normalized against Gapdh, n = 5). [file Image_1.TIF]

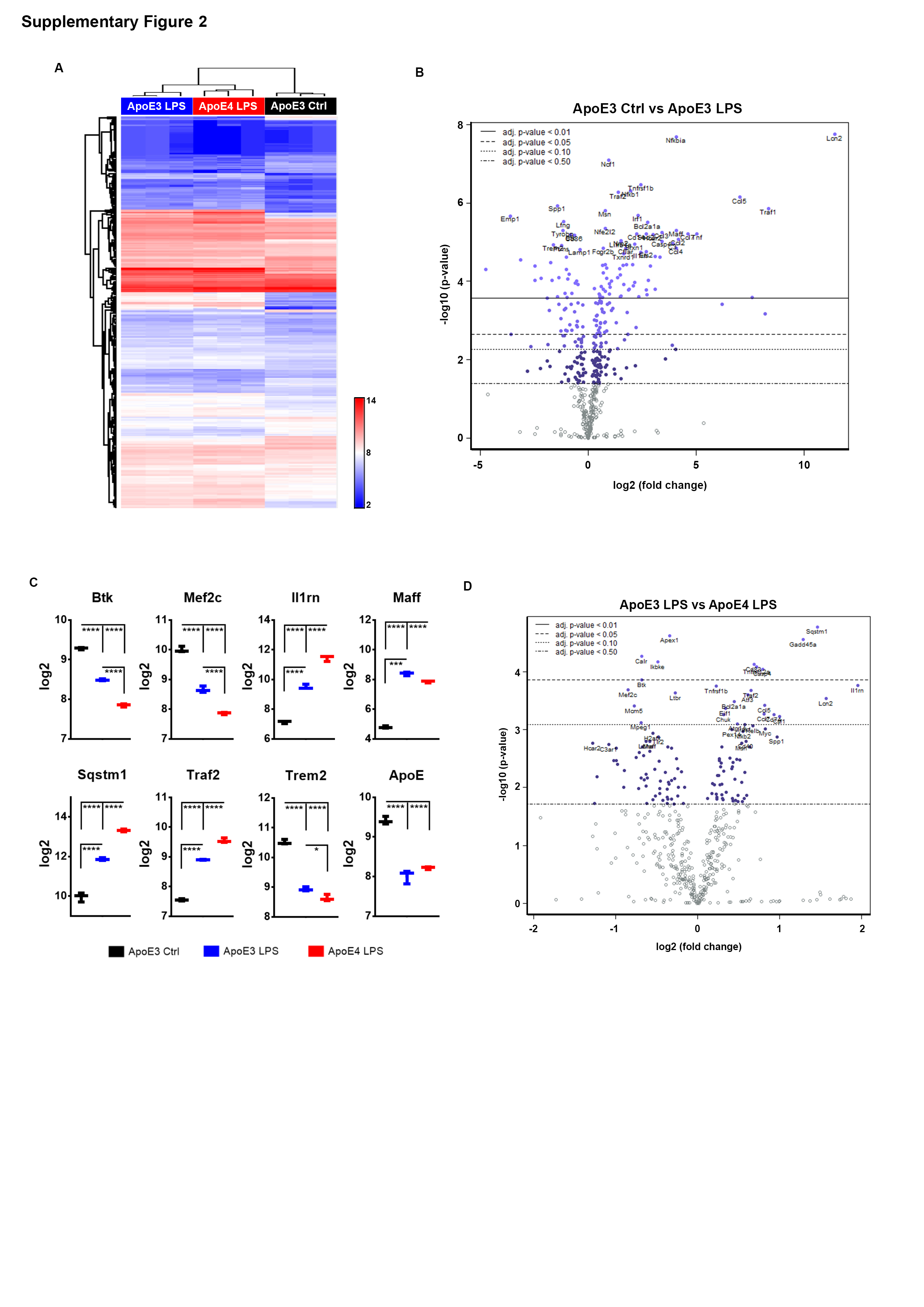

Supplement: FIGURE S2 — Proinflammatory signaling is altered in N9.ApoE4 after LPS treatment. The NanoString nCounter Mouse Neuroinflammation Panel was used to evaluate gene expression analysis of inflammation-relevant genes, in N9.ApoE3 and N9.ApoE4 after 24 h of treatment with 100 ng/ml LPS. (A) Heatmap depicting cluster analysis for gene expression levels of 389 out of the 770 genes included in the panel that presented differential expression (upregulated red, downregulated blue) between N9.ApoE3 cells as controls (n = 3, black bar) and LPS treated ApoE3 (n = 3 blue bar) and ApoE4 (n = 3, red bar) N9 cells. (B) Volcano plot for t-test pairwise analysis between LPS treated and control N9.ApoE3 cells. 152 genes showed to be differentially regulated between treated N9.ApoE3 cells and controls after adjusting for false discovery rate (FDR) using the Benjamini–Yekutieli procedure (see Supplementary Table 1). (C) Boxplots displaying significantly different expression in LPS treated and untreated ApoE3/4 N9 cells after Two-way ANOVA analysis. Expression levels of Bruton tyrosine kinase (Btk), Myocyte Enhancer Factor 2C (MEF2c) and Triggering receptor expressed on myeloid cells 2 (Trem2) are significantly decreased in LPS treated cells, particularly in N9.ApoE4 treated cells. Conversely, expression levels of Interleukin-1 receptor antagonist (Il1rn), Sequestosome-1 (Sqstm1) and TNF receptor-associated factor 2 (Traf2), showed to be significantly upregulated after LPS treatment, with higher expression levels in N9.ApoE4 treated cells. This trend was not observed in Transcription factor MafF (Maff) expression levels, in which, although significantly upregulated in ApoE transfected N9 cells after LPS treatment, N9.ApoE4 cells expressed significantly lower levels than N9.ApoE3 cells. ApoE expression levels of the endogenous mouse ApoE are depicted as controls, with LPS treated cells showing significantly lower levels, (n = 3, see also Supplementary Table 2). (D) Volcano plot for t-test pairwise ana [file Image_2.TIF]

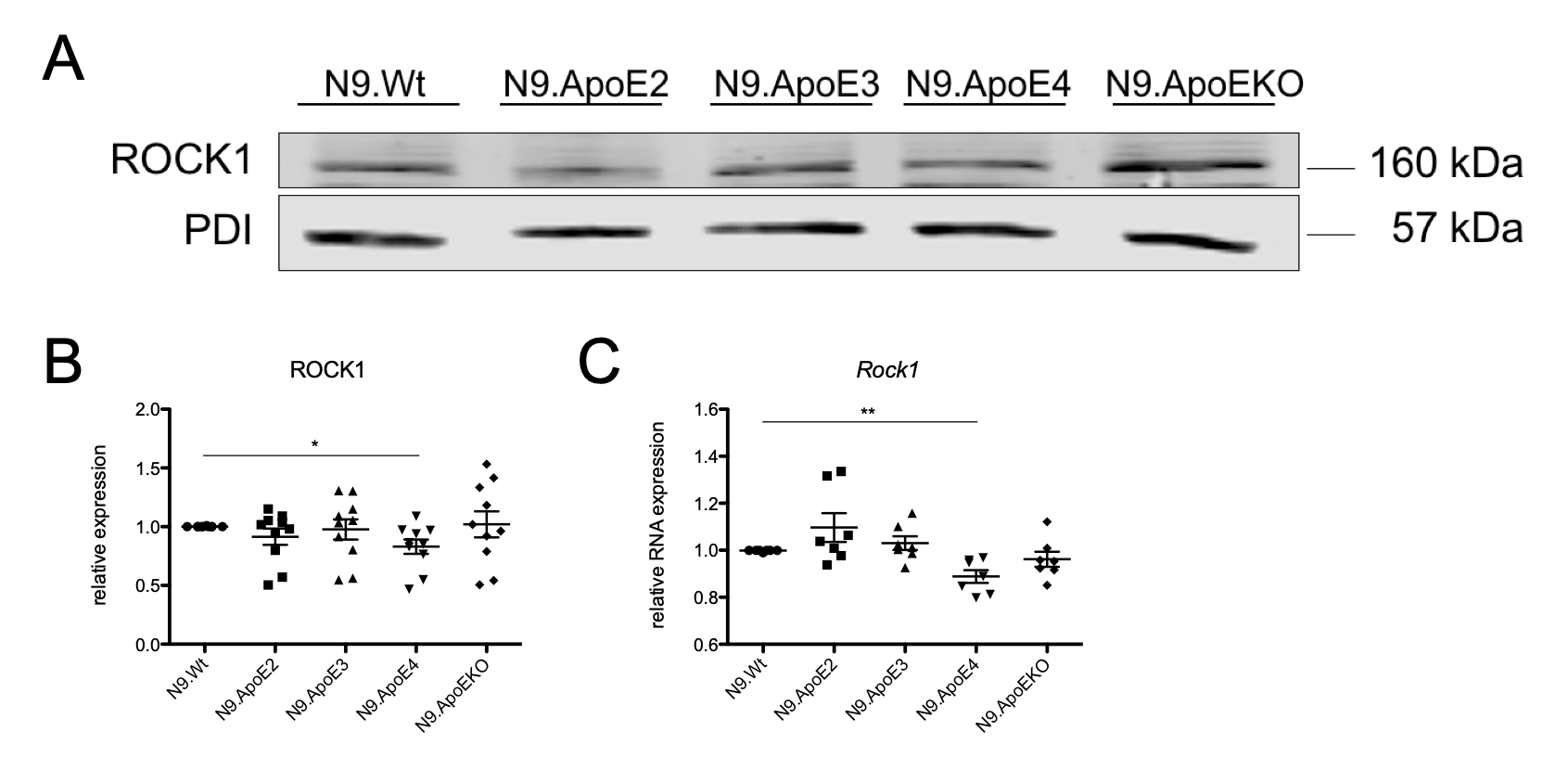

Supplement: FIGURE S3 — ROCK1 expression is dysregulated in N9.ApoE4. (A) Western Blot analysis of ROCK1. PDI displays protein loading. (B) Quantification shows a significant downregulation of ROCK1 in N9.ApoE4 in comparison to N9.Wt (n = 9). (C) Quantification of RNA-expression level of Rock1 shows a significant decrease in N9.ApoE4 in comparison to N9.Wt (n = 7). Murine GapDH were used as endogenous control. [file Image_3.TIF]
