# Supplementary material for: Phagocytosis of Apoptotic Cells Is Specifically Upregulated in ApoE4 Expressing Microglia in vitro
Source: Front Cell Neurosci. 2019 May 3;13:181. doi: 10.3389/fncel.2019.00181 (PMC6509203; doi:10.3389/fncel.2019.00181)
Supplement: TABLE S1 — Pairwise analysis of gene expression levels between untreated N9.ApoE3, and LPS treated N9.ApoE3 cells. [file Table_1.DOCX]

**Supplementary Table 1.** **Pairwise analysis of gene expression levels between untreated N9.ApoE3 and LPS treated N9.ApoE3 cells**

| **Accession Number** | **Probe Name** | **Log2 fold change** | **Standard error (log2)** | **p value** | **Benjamin-Yekutieli Adjusted p value** |
| --- | --- | --- | --- | --- | --- |
| NM_008491.1 | Lcn2 | 11.40 | 0.084 | 0.000000018 | 0.0000338 |
| NM_010907.2 | Nfkbia | 4.09 | 0.031 | 0.000000020 | 0.0000338 |
| NM_001286037.1 | Ncf1 | 0.97 | 0.010 | 0.000000082 | 0.0000916 |
| NM_011610.3 | Tnfrsf1b | 2.43 | 0.038 | 0.000000342 | 0.0002880 |
| NM_008689.2 | Nfkb1 | 1.96 | 0.033 | 0.000000492 | 0.0003020 |
| NM_009422.2 | Traf2 | 1.38 | 0.024 | 0.000000539 | 0.0003020 |
| NM_013653.1 | Ccl5 | 7.03 | 0.130 | 0.000000710 | 0.0003410 |
| NM_009263.3 | Spp1 | -1.42 | 0.030 | 0.000001160 | 0.0004880 |
| NM_009421.3 | Traf1 | 8.35 | 0.183 | 0.000001370 | 0.0005120 |
| NM_010833.2 | Msn | 0.78 | 0.018 | 0.000001550 | 0.0005220 |
| NM_008390.1 | Irf1 | 2.30 | 0.056 | 0.000002070 | 0.0005970 |
| NM_010128.4 | Emp1 | -3.61 | 0.088 | 0.000002130 | 0.0005970 |
| NM_008494.3 | Lfng | -1.13 | 0.030 | 0.000003030 | 0.0007520 |
| NM_009742.3 | Bcl2a1a | 2.74 | 0.074 | 0.000003130 | 0.0007520 |
| NR_132727.1 | Nfe2l2 | 0.80 | 0.024 | 0.000004400 | 0.0008870 |
| NM_010755.3 | Maff | 4.07 | 0.123 | 0.000005010 | 0.0008870 |
| NM_011662.2 | Tyrobp | -1.18 | 0.036 | 0.000005100 | 0.0008870 |
| NM_011337.1 | Ccl3 | 3.42 | 0.106 | 0.000005580 | 0.0008870 |
| NM_013654.3 | Ccl7 | 4.62 | 0.147 | 0.000006030 | 0.0008870 |
| NM_013671.3 | Sod2 | 2.68 | 0.085 | 0.000006060 | 0.0008870 |
| NM_009841.3 | Cd14 | 2.26 | 0.072 | 0.000006080 | 0.0008870 |
| NM_013693.2 | Tnf | 5.03 | 0.160 | 0.000006080 | 0.0008870 |
| NM_013482.2 | Btk | -0.81 | 0.026 | 0.000006350 | 0.0008870 |
| NM_030701.1 | Hcar2 | 3.02 | 0.097 | 0.000006420 | 0.0008870 |
| NM_019388.3 | Cd86 | -0.62 | 0.020 | 0.000006590 | 0.0008870 |
| NM_011333.3 | Ccl2 | 4.18 | 0.144 | 0.000008500 | 0.0011000 |
| NM_001077403.1 | Nrp2 | 1.51 | 0.053 | 0.000009000 | 0.0011100 |
| NM_007609.2 | Casp4 | 3.43 | 0.121 | 0.000009410 | 0.0011100 |
| NM_013532.3 | Lilrb4a | 1.51 | 0.054 | 0.000009530 | 0.0011100 |
| XM_011246258.1 | C3 | 3.92 | 0.143 | 0.000010600 | 0.0011900 |

* Top 30 genes

**Supplementary Table 2. Two-way ANOVA analysis of gene expression levels between untreated N9.ApoE3, LPS treated N9.ApoE3 and LPS treated N9.ApoE4 cells**

| **Accession Number** | **Probe Name** | **Two-way ANOVA p value** | | |
| --- | --- | --- | --- | --- |
|  |  | **Haplotype** | **Treatment** | **Interaction** |
| NM_013653.1 | Ccl5 | 0.0000000417 | 0.0000000030 | 0.0000000944 |
| NM_011610.3 | Tnfrsf1b | 0.0000000974 | 0.0000000059 | 0.0000001840 |
| NM_009742.3 | Bcl2a1a | 0.0000007070 | 0.0000000619 | 0.0000018700 |
| NM_013693.2 | Tnf | 0.0000031800 | 0.0000000618 | 0.0000019400 |
| NM_010907.2 | Nfkbia | 0.0000036200 | 0.0000000713 | 0.0000022300 |
| NM_013482.2 | Btk | 0.0000001820 | 0.0000001290 | 0.0000023300 |
| NM_010755.3 | Maff | 0.0000120000 | 0.0000000953 | 0.0000028600 |
| NM_009422.2 | Traf2 | 0.0000004750 | 0.0000001470 | 0.0000035500 |
| NM_013654.3 | Ccl7 | 0.0000015500 | 0.0000001470 | 0.0000043900 |
| NM_009841.3 | Cd14 | 0.0000023800 | 0.0000001720 | 0.0000052500 |
| NM_007609.2 | Casp4 | 0.0000020400 | 0.0000002580 | 0.0000074200 |
| NM_008491.1 | Lcn2 | 0.0000024900 | 0.0000002590 | 0.0000076500 |
| NM_013532.3 | Lilrb4a | 0.0000037200 | 0.0000003360 | 0.0000100000 |
| NM_011337.1 | Ccl3 | 0.0000075400 | 0.0000003310 | 0.0000103000 |
| NM_013671.3 | Sod2 | 0.0000233000 | 0.0000003430 | 0.0000105000 |
| NM_011611.2 | Cd40 | 0.0000038800 | 0.0000004120 | 0.0000121000 |
| NM_207653.3 | Cflar | 0.0000061600 | 0.0000005630 | 0.0000167000 |
| NM_011809.2 | Ets2 | 0.0000047400 | 0.0000006120 | 0.0000174000 |
| NM_031167.5 | Il1rn | 0.0000014400 | 0.0000010200 | 0.0000182000 |
| NM_029688.4 | Srxn1 | 0.0000076500 | 0.0000008460 | 0.0000245000 |
| NM_011333.3 | Ccl2 | 0.0000192000 | 0.0000009290 | 0.0000285000 |
| XM_011246258.1 | C3 | 0.0000285000 | 0.0000009630 | 0.0000297000 |
| NM_001170537.1 | Mef2c | 0.0000042100 | 0.0000018200 | 0.0000387000 |
| NM_011018.2 | Sqstm1 | 0.0000038100 | 0.0000024600 | 0.0000450000 |
| NM_001113553.1 | Irak2 | 0.0000196000 | 0.0000016500 | 0.0000486000 |
| NM_010554.4 | Il1a | 0.0000422000 | 0.0000016000 | 0.0000489000 |
| NM_015762.2 | Txnrd1 | 0.0000231000 | 0.0000016800 | 0.0000499000 |
| NM_008689.2 | Nfkb1 | 0.0000367000 | 0.0000017200 | 0.0000522000 |
| NM_008390.1 | Irf1 | 0.0000159000 | 0.0000018800 | 0.0000534000 |
| NM_013652.1 | Ccl4 | 0.0000280000 | 0.0000020100 | 0.0000596000 |
| NM_031254.2 | Trem2 | 0.0000213000 | 0.0000021800 | 0.0000625000 |
| NM_001113530.1 | Csf1 | 0.0000061300 | 0.0000033300 | 0.0000648000 |
| NM_027817.3 | Grap | 0.0000487000 | 0.0000029200 | 0.0000868000 |

* Top 33 genes, all with p<0.0001 values after post hoc Tukey’s HSD test.

**Supplementary Table 3. Pairwise analysis of gene expression levels between LPS treated N9.ApoE3 and N9.ApoE4 cells**

| **Accession Number** | **Probe name** | **Log2 fold change** | **Standard error (log2)** | **p value** | **Benjamin-Yekutieli Adjusted p value** |
| --- | --- | --- | --- | --- | --- |
| NM_011018.2 | Sqstm1 | 1.46 | 0.0599 | 0.000017 | 0.0303 |
| NM_009687.2 | Apex1 | -0.344 | 0.0154 | 0.000024 | 0.0303 |
| NM_007836.1 | Gadd45a | 1.29 | 0.0599 | 0.000028 | 0.0303 |
| NM_007591.3 | Calr | -0.684 | 0.0376 | 0.000054 | 0.0372 |
| NM_019777.3 | Ikbke | -0.485 | 0.0281 | 0.000066 | 0.0372 |
| NM_009856.2 | Cd83 | 0.691 | 0.0413 | 0.000075 | 0.0372 |
| NM_001161746.1 | Tnfrsf12a | 0.719 | 0.0439 | 0.000081 | 0.0372 |
| NM_007609.2 | Casp4 | 0.795 | 0.0499 | 0.000091 | 0.0372 |
| NM_013482.2 | Btk | -0.685 | 0.0476 | 0.000136 | 0.0497 |
